# Supplementary material for: Morphology, phylogeny, and taxonomy of two species of colonial volvocine green algae from Lake Victoria, Tanzania
Source: PLoS One. 2019 Nov 11;14(11):e0224269. doi: 10.1371/journal.pone.0224269 (PMC6844456; doi:10.1371/journal.pone.0224269)
Supplement: S1 Fig — (DOCX) [file pone.0224269.s001.docx]

**
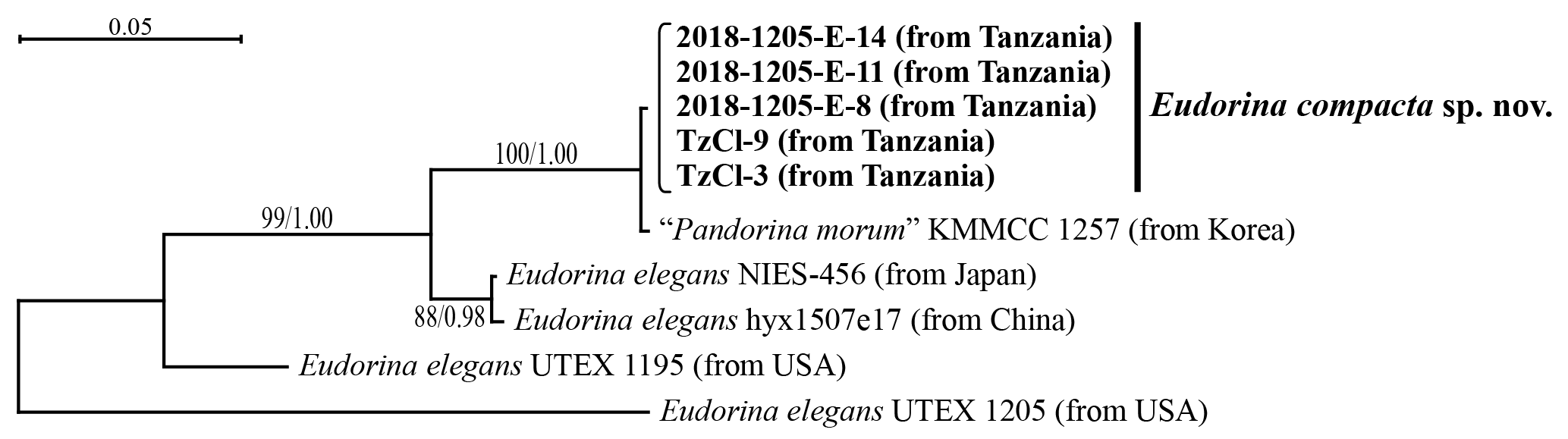
**

**S1 Fig. Maximum likelihood (ML) tree of *Eudorina* species related to *Eudorina compacta* from Lake Victoria based on ITS region of nuclear ribosomal DNA (ITS-1, 5.8S rDNA, and ITS-2) (S1 Table).**

Branch lengths are proportional to the evolutionary distances that are indicated by the scale bar above the tree. Bootstrap values from ML (left) and posterior probabilities from Bayesian (right) analyses are shown on the branches.
