# Supplementary material for: Morphology, phylogeny, and taxonomy of two species of colonial volvocine green algae from Lake Victoria, Tanzania
Source: PLoS One. 2019 Nov 11;14(11):e0224269. doi: 10.1371/journal.pone.0224269 (PMC6844456; doi:10.1371/journal.pone.0224269)
Supplement: S5 Fig — (DOCX) [file pone.0224269.s005.docx]

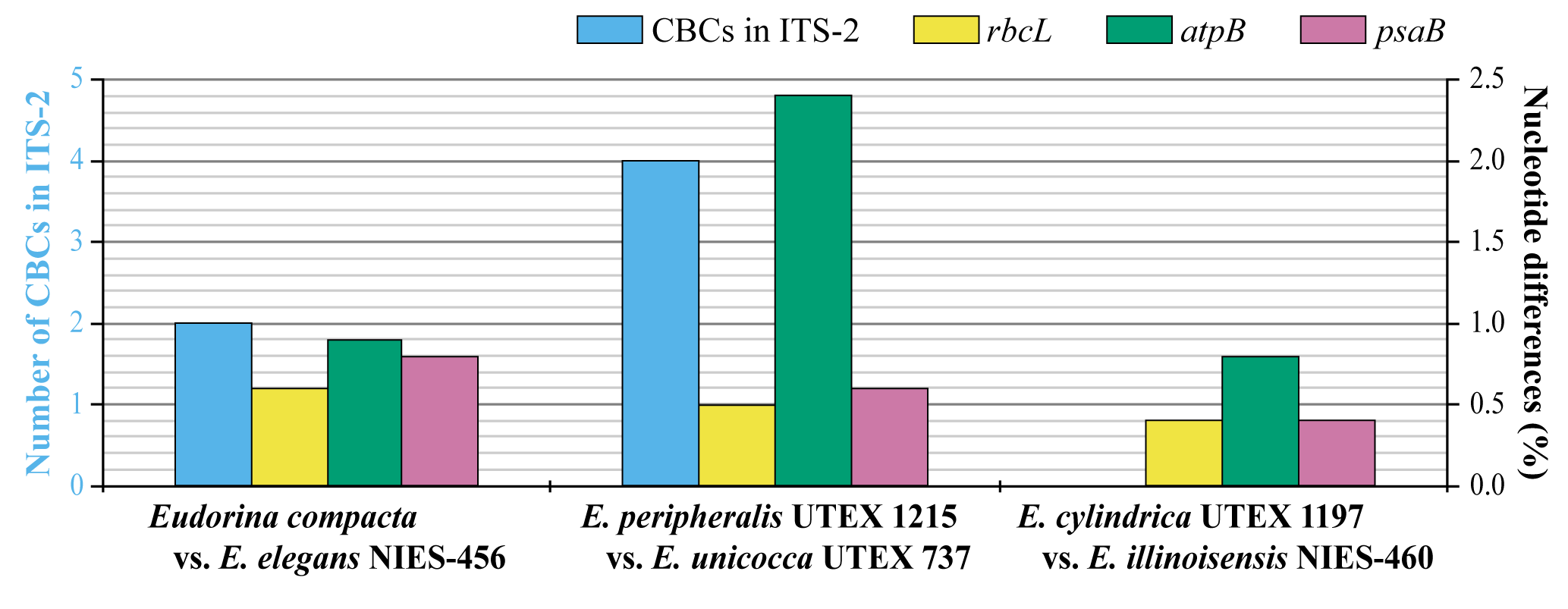


**S5 Fig. Comparison of genetic distances between closely related strains in the genus *Eudorina*.**

Morphological and genetic differences between *E. unicocca* (including strain UTEX 737) and *E. peripheralis* (including UTEX 1215) were already demonstrated [1,2]. Morphological difference and genetic isolation between *E. cylindrica* strain UTEX 1197 and *E. illinoisensis* strain NIES-460 are apparent based on the previous studies [1,3-5]. Comparisons of ITS-2 secondary structures to detect compensatory base changes (CBCs) are shown in S2, S6 and S7 Figures. Strains and accession numbers of sequences analyzed (*rbcL*, *atpB*, *psaB* and ITS-2 rDNA, respectively) are as follows: *E. compacta* strain 2018-1205-E14 (LC504545, LC504553, LC504554 and LC504534), *E. elegans* strain NIES-456 (D63432, AB014009, AB044435 and LC504542), *E. peripheralis* strain UTEX 1215 (D63434, AB014007, AB044440 and AF486525), *E. unicocca* strain UTEX 737 [= NIES-724] (D86829, AB014008, AB044439, and AB359069/AF100901), *E. cylindrica* strain UTEX 1197 (D86833, AB014033, AB044441 and HG422756) and *E. illinoisensis* strain NIES-460 (D63433, AB014013, AB044434 and LC504544).

**References**

1. Goldstein M. Speciation and mating behavior in *Eudorina*. J Protozool. 1964; 11: 317–344. doi: 10.1111/j.1550-7408.1964.tb01762.x.
2. Yamada TK, Miyaji K, Nozaki H. A taxonomic study of *Eudorina unicocca* (Volvocaceae, Chlorophyceae) and related species, based on morphology and molecular phylogeny. Eur J Phycol. 2018; 43: 317-326. doi: 10.1080/09670260701763484.
3. Nozaki H. Notes on microalgae in Japan (10). *Eudorina illinoisensis* (Chlorophyta， Volvocales). Jpn J Phycol. 1986; 34: 144 (In Japanese).
4. Nozaki H, Krienitz L. Morphology and phylogeny of *Eudorina minodii* (Chodat) Nozaki et Krienitz, comb. nov. (Volvocales, Chlorophyta) from Germany. Eur J Phycol. 2001; 36: 23–28. doi: 10.1080/09670260110001735158.
5. Coleman AW. Comparison of *Eudorina*/*Pleodorina* ITS sequences of isolates from nature with those from experimental hybrids. Am J Bot. 2002; 89:1523-1530. doi: 10.3732/ajb.89.9.1523.
