# Supplementary material for: Morphology, phylogeny, and taxonomy of two species of colonial volvocine green algae from Lake Victoria, Tanzania
Source: PLoS One. 2019 Nov 11;14(11):e0224269. doi: 10.1371/journal.pone.0224269 (PMC6844456; doi:10.1371/journal.pone.0224269)
Supplement: S7 Fig — (DOCX) [file pone.0224269.s007.docx]

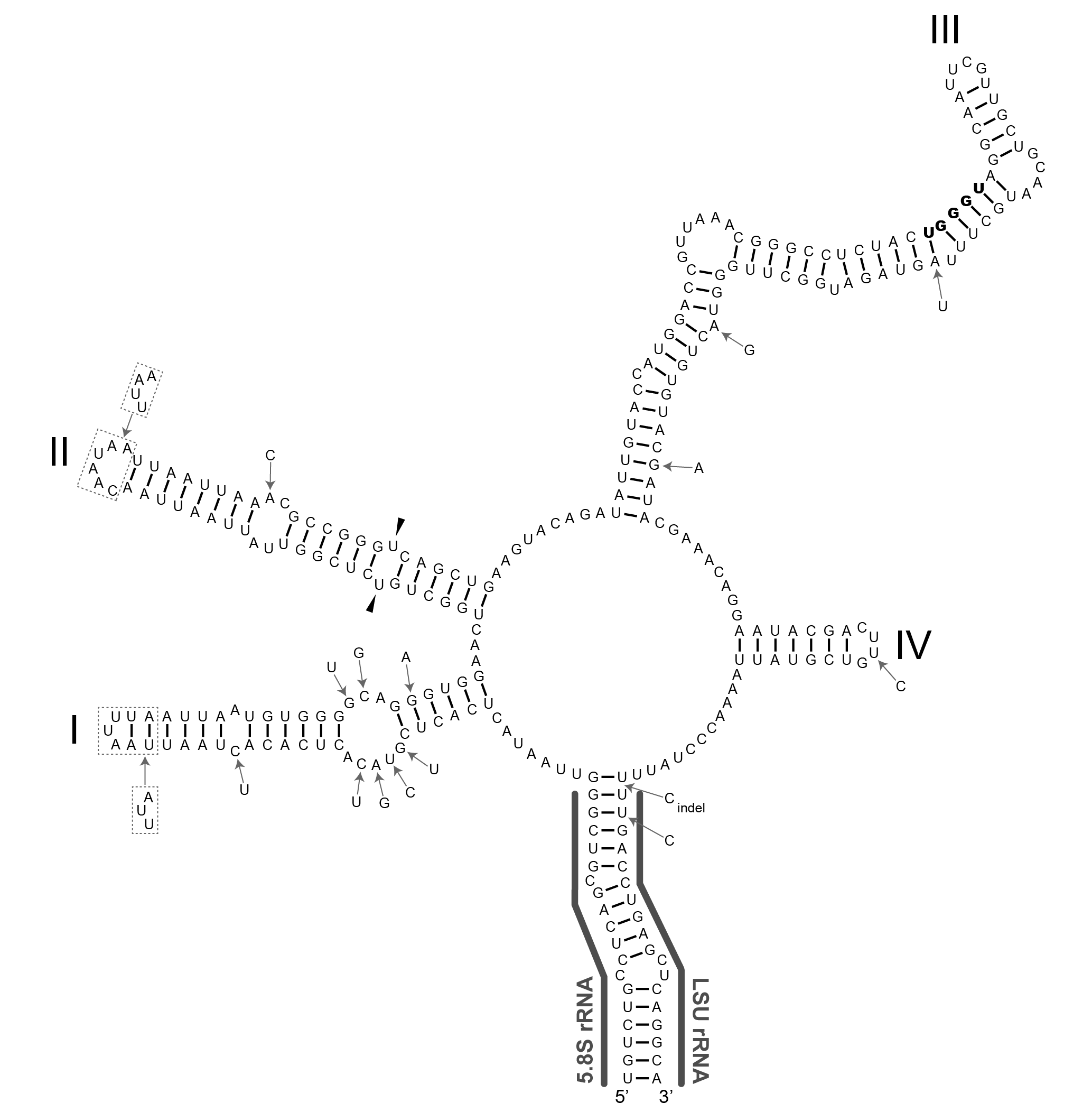


**S7 Fig. The secondary structure of nuclear ribosomal DNA (rDNA) internal transcribed spacer 2 (ITS-2) transcript of *Eudorina cylindrica* strain UTEX 1197, including the 3’ end of the 5.8S ribosomal RNA (RNA) and the 5’ end of the LSU rRNA.**

Secondary structure of nuclear rDNA ITS-2 was drawn using VARNA version 3.9. Note the U-U mismatch in helix II (arrowheads) and the YGGY motif on the 5’ side near the apex of helix III (boldface), common structural hallmarks of eukaryotic nuclear rDNA ITS-2 secondary structures. Nucleotide differences between *E. cylindrica* strain UTEX 1197 and *E. illinoisensis* strain NIES-460 are shown by arrows.
