# Supplementary material for: Morphology, phylogeny, and taxonomy of two species of colonial volvocine green algae from Lake Victoria, Tanzania
Source: PLoS One. 2019 Nov 11;14(11):e0224269. doi: 10.1371/journal.pone.0224269 (PMC6844456; doi:10.1371/journal.pone.0224269)
Supplement: S1 Table — (DOCX) [file pone.0224269.s008.docx]

## S1 Table. List of colonial volvocine species/strains included in the phylogenetic analyses of ITS-2 sequences (with DDBJ/EMBL/GENBANK accession numbers; S1 and S3 Figs).

| Species | Strain designation | Origin | Gender^1^ | Accession no. | Reference |
| --- | --- | --- | --- | --- | --- |
| *Eudorina compacta* | 2018-1205-E-14 (=NIES^2^-4373) | Sample 1-05^3^, Lake Victoria | Male | LC504534^4^ | The present study |
| *Eudorina compacta* | 2018-1205-E-11 (=NIES-4374) | Sample 1-05, Lake Victoria | Male | LC504534^4^ | The present study |
| *Eudorina compacta* | 2018-1205-E-8 (=NIES-4375) | Sample 1-05, Lake Victoria | Male | LC504536^4^ | The present study |
| *Eudorina compacta* | TzCl-9 (=NIES-4376) | Sample 8-2^5^, Lake Victoria | Female | LC504537^4^ | The present study |
| *Eudorina compacta* | TzCl-3 (=NIES-4377) | Sample 1-03^6^, Lake Victoria | Female | LC504538^4^ | The present study |
| *Eudorina elegans* | NIES-456 | Tokyo, Japan | Male | LC504542^4^ | [1] |
| *Eudorina elegans* | UTEX^7^ 1195 | USA | ND^8^ | LC504543^4^ | [2] |
| *Eudorina elegans* | UTEX 1205 | USA | ND | HG422761 | [2] |
| *Eudorina elegans* | hyx1507e17 | China | ND | KX247722 |  |
| “*Pandorina morum*” | KMMCC 1257 | Korea | ND | JQ315555 |  |
| *Colemanosphaera charkowiensis* | 2018-1204-C-1 (=NIES-4378) | Sample 8-2, Lake Victoria | ND | LC504539^4^ | The present study |
| *Colemanosphaera charkowiensis* | 2018-1205-C-7 (=NIES-4379) | Sample 1-05, Lake Victoria | ND | LC504540^4^ | The present study |
| *Colemanosphaera charkowiensis* | 2018-1205-C-13 (=NIES-4380) | Sample 1-05, Lake Victoria | ND | LC504541^4^ | The present study |
| *Colemanosphaera charkowiensis* | Isa7-1 (=NIES-3383) | Lake Isanuma, Japan | ND | AB905583 | [3] |
| *Colemanosphaera charkowiensis* | 2013-0615-IC-3 (=NIES-3386) | Lake Isanuma, Japan | ND | AB905582 | [3] |
| *Colemanosphaera angeleri* | 2010-1206-1 (NIES-3382) | Lake Isanuma, Japan | ND | AB905586 | [3] |
| *Colemanosphaera angeleri* | ASW05157 | Austria | ND | AF182439 | [3] |
| *Platydorina caudata* | UTEX 1661 | USA | ND | AB905587 | [2] |

^1^ Based on presence (male) or absence (female) of minus dominance *MID* gene by genomic PCR.

^2^ Microbial Culture Collection at the National Institute for Environmental Studies [4].

^3^ Water sample 1-05 (pH 9.5; 25.6 °C) of Lake Victoria was collected at the pier of TAFIRI Mwanza Centre, Mwanza, Tanzania (02°34'53.43''S, 032°53'43.76''E), on 5 December 2018.

^4^ Sequenced in the present study.

^5^ Water sample 8-2 (pH 9.7; 26.0 °C) of Lake Victoria was collected at a small island within Mwanza Bay, Mwanza, Tanzania (02°34'07.83''S, 032°53'37.77''E), on 4 December 2018.

^6^ Water sample 1-03 (pH 10.6; 26.7 °C) of Lake Victoria was collected at the pier of TAFIRI Mwanza Centre, Mwanza, Tanzania (02°34'53.43''S, 032°53'43.76''E), on 3 December 2018.

^7^ Culture Collection of Algae at the University of Texas at Austin [2].

^8^ Not detected.

**References**

1. Nozaki H. Sexual reproduction in *Eudorina elegans* (Chlorophyta, Volvocales). Bot Mag Tokyo 1983; 96: 103-110.
2. Starr RC, Zeikus JA. UTEX - The Culture Collection of Algae at the University of Texas at Austin. J Phycol. 1993; 29 (2), Supplement: 1-106.
3. Nozaki H, Yamada TK, Takahashi F, Matsuzaki R, Nakada T. New ‘missing link’ genus of the colonial volvocine green algae gives insights into the evolution of oogamy. BMC Evol Biol. 2014; 14: 37. doi: 10.1186/1471-2148-14-37
4. Kawachi M, Ishimoto M, Mori F, Yumoto K, Sato M, Noël M-H. MCC-NIES list of strains, 9th Edition, microbial culture collection at National Institute for Environmental Studies, Tsukuba, Japan; 2013. <http://mcc.nies.go.jp/download/list9th_e.pdf>
