# Supplementary material for: Morphology, phylogeny, and taxonomy of two species of colonial volvocine green algae from Lake Victoria, Tanzania
Source: PLoS One. 2019 Nov 11;14(11):e0224269. doi: 10.1371/journal.pone.0224269 (PMC6844456; doi:10.1371/journal.pone.0224269)
Supplement: S2 Table — (DOCX) [file pone.0224269.s009.docx]

**S2 Table. Primers used for genomic PCR of possible male-specific minus dominance (*MID*) gene of *Eudorina compacta*.**

| Designation | Sequence (5’–3’) |
| --- | --- |
| N456E15MID-F | CAGAACACKGAATGGCTKAARGAGTGCAT |
| N456E15MID-R^a^ | CTTTCTGTAMGGCCATCKTGGKATCCC |

^a^ Reverse primer.
