# Supplementary material for: Morphology, phylogeny, and taxonomy of two species of colonial volvocine green algae from Lake Victoria, Tanzania
Source: PLoS One. 2019 Nov 11;14(11):e0224269. doi: 10.1371/journal.pone.0224269 (PMC6844456; doi:10.1371/journal.pone.0224269)
Supplement: S3 Table — (DOC) [file pone.0224269.s010.doc]

**S3 Table. List of the colonial volvocine taxa/strains included in the phylogenetic analysis (Fig. 1) and DDBJ/EMBL/GenBank accession numbers of *rbcL*** genes.

| Taxon | Strain designation | DDBJ/EMBL/GenBank accession number |
| --- | --- | --- |
| *Pandorina morum* | UTEX 2326 | AB044164 |
| *Pandorina colemaniae* | NIES-572 | D63441 |
| *Volvulina pringsheimii* | UTEX 1020 | D63444d |
| *Volvulina steinii* | NIES-545 | AB044159 |
| *Yamagishiella unicocca* | UTEX 2428 | D86823 |
| *Yamagishiella unicocca* | UTEX 2430 | D86825f |
| *Yamagishiella unicocca* | NIES-872 | AB044168 |
| *Platydorina caudata* | UTEX 1658 | D86828 |
| *Colemanosphaera charkowiensis* | 2018-1204-C-1 (=NIES-4378) | LC504550^1^ |
| *Colemanosphaera charkowiensis* | 2018-1205-C-7 (=NIES-4379) | LC504551^1^ |
| *Colemanosphaera charkowiensis* | 2018-1205-C-13 (=NIES-4380) | LC504552^1^ |
| *Colemanosphaera charkowiensis* | Isa 7-1 (=NIES-3383) | AB905591 |
| *Colemanosphaera angeleri* | 2010-0126-1 (=NIES-3382) | AB905592 |
| *Eudorina cylindrica* | UTEX 1197 | D86833 |
| *Eudorina peripheralis* | UTEX 1215^c^ | D63434 |
| *Eudorina unicocca* | UTEX 737 | D86829 |
| *Eudorina elegans* | NIES-456 | D63432 |
| *Eudorina elegans* | UTEX 1205 | D88805 |
| *Eudorina elegans* | UTEX 1212 | D88806 |
| *Eudorina elegans* | UTEX 1195 | D88810 |
| *Eudorina elegans* | UTEX 1199 | D88804 |
| *Eudorina* sp. from Taiwan | 2006-703-Eu-15 (=NIES-2735) | AB458679 |
| *Eudorina minodii* | NIES-856 | AB047074, AB47075, AB47076 |
| *Eudorina compacta* | 2018-1205-E-14 (=NIES^2^-4373) | LC504545^1^ |
| *Eudorina compacta* | 2018-1205-E-11 (=NIES-4374) | LC504546^1^ |
| *Eudorina compacta* | 2018-1205-E-8 (=NIES-4375) | LC504547^1^ |
| *Eudorina compacta* | TzCl-9 (=NIES-4376) | LC504548^1^ |
| *Eudorina compacta* | TzCl-3 (=NIES-4377) | LC504549^1^ |
| *Eudorina illinoisensis* | NIES-460 | D63433 |
| *Pleodorina thompsonii* | UTEX 2804 | AB214408 |
| *Pleodorina starrii* | NIES-1362 | AB214427 |
| *Pleodorina indica* | UTEX 1990 | D86834 |
| *Pleodorina japonica* | UTEX 2523 | D63440 |
| *Pleodorina californica* | UTEX 809 | D63439 |
| *Pleodorina sphaerica* | 2015-1128-2P-12 (=NIES-4066) | LC215634 |
| *Volvox africanus* | 2013-0703-VO4 (=NIES-3780) | LC090149 |
| *Volvox gigas* | UTEX 1895 | AB076084 |
| *Volvox ovalis* | NIES-2569 | AB592342 |
| *Volvox obversus* | UTEX 1865 | AB076085 |
| *Volvox reticuliferus* | UTEX 1891 | AB076101 |
| *Volvox reticuliferus* | 2013-0703-VO2 (=NIES-3782) | LC090154 |
| *Volvox tertius* | UTEX 132 | AB076098 |
| *Volvox tertius* | NIES-544 | AB086174 |
| *Volvox powersii* | UTEX 1863 | AB214415 |
| *Volvox carteri* |  |  |
| f. *kawasakiensis* | NIES-732 | D63446 |
| f. *nagariensis* | UTEX 1885 | AB076099 |
| f. *weismannia* | UTEX 1875 | AB076100 |
| *Volvox aureus* | NIES-541 | D63445 |
| *Volvox aureus* | NIES-891 | AB076096 |
| *Volvox aureus* | NIES-892 | AB076086 |
| *Volvox zeikusii* | UTEX 2184 (=NIES-731) | D63447 |
| *Volvox dissipatrix* | Marb.2RS 29 | AB214420 |
| *Volvox rousseletii* | UTEX 1862 | D63448 |
| *Volvox barberi* | UTEX 804 | D86835 |
| *Volvox globator* | UTEX 955 | D86836 |

^1^Sequenced in the present study.
