# Supplementary material for: Morphology, phylogeny, and taxonomy of two species of colonial volvocine green algae from Lake Victoria, Tanzania
Source: PLoS One. 2019 Nov 11;14(11):e0224269. doi: 10.1371/journal.pone.0224269 (PMC6844456; doi:10.1371/journal.pone.0224269)
Supplement: Attachment — Submitted filename: Response to Reviewers.docx [file pone.0224269.s011.docx]

Dear Dr. Jim Umen,

Thank you very much for your positive review results of our manuscript entitled "Morphology, phylogeny and taxonomy of two species of colonial volvocine green algae from Lake Victoria, Tanzania " submitted for publication in ***PLOS ONE*** as a research article.

Based on the comments raised by Reviewers the academic editor, the manuscript has been revised adequately. Accession numbers of the new sequences, study ID of the rDNA ITS regions and *rbcL*-*psbC* sequences used for construction of the phylogenetic tress (Fig. 1 and S1 and S3 Figs) and NIES strain designations of the new Tanzanian strains of *Eudorina compacta* and *Colemanosphaera charkowiensis* have been obtained and described in the revised manuscript.

Our responses to the comments by the academic editor and Reviewers have been described in red below.

PONE-D-19-26214
Morphology, phylogeny, and taxonomy of two species of colonial volvocine green algae from Lake Victoria, Tanzania
PLOS ONE

Dear Dr. Nozaki,

Thank you for submitting your manuscript to PLOS ONE. After careful consideration, we feel that it has merit but does not fully meet PLOS ONE’s publication criteria as it currently stands. Therefore, we invite you to submit a revised version of the manuscript that addresses the points raised during the review process.

I am requesting some minor changes as described below.

In your revision please consider the suggestions of Reviewer 1 including the request for inclusion of more information on biodiversity and geographical ranges.  The significance of the collection site being an ancient lake (versus another type of lake or aquatic habitat) was not clear, so if you leave the “ancient lake” description in the manuscript please add some additional information to provide context on why this is an important distinction to make.

**Response: In order to explain the significance of the collection site being an ancient lake, the following three references ([6-8]) suggesting the importance of ancient lakes worldwide in the biodiversity studies have been cited in the Introduction section of the revised manuscript.**

**6. Martens K. Speciation in ancient lakes. Trends Ecol Evol. 1997; 12: 177–182. doi: 10.1016/s0169-5347(97)01039-2.**

**7. Cristescu ME, Adamowicz SJ, Vaillant JJ, Haffner DG. Ancient lakes revisited: from the ecology to the genetics of speciation. Mol Ecol. 2010; 19: 4837–4851. doi: 10.1111/j.1365-294X.2010.04832.x.**

**8. Kulikovskiy MS, Lange-Bertalot H, Kuznetsova IV. Lake Baikal: hotspot of endemic diatoms II. In: Lange-Bertalot H. editor. Iconographia Diatomologica Volume: 26. Oberreifenberg: Koeltz Scientific Books; 2015. pp. 1-656.**

For Reviewer 2, Point 1, PLoS ONE does not discriminate based on perceived impact or target audience size, so no need to address this comment.  Point 2 raises a valid issue that continues to complicate taxonomic classification of volvocine algae due to extensive polyphyly, but I don’t feel it is something that must be solved here in order to publish this work.

**Response: I agree with these AE comments. See Responses described below.**

We would appreciate receiving your revised manuscript by Nov 16 2019 11:59PM. When you are ready to submit your revision, log on to https://www.editorialmanager.com/pone/ and select the 'Submissions Needing Revision' folder to locate your manuscript file.

If you would like to make changes to your financial disclosure, please include your updated statement in your cover letter.

To enhance the reproducibility of your results, we recommend that if applicable you deposit your laboratory protocols in protocols.io, where a protocol can be assigned its own identifier (DOI) such that it can be cited independently in the future. For instructions see: <http://journals.plos.org/plosone/s/submission-guidelines#loc-laboratory-protocols>

Please include the following items when submitting your revised manuscript:

- A rebuttal letter that responds to each point raised by the academic editor and reviewer(s). This letter should be uploaded as separate file and labeled 'Response to Reviewers'.
- A marked-up copy of your manuscript that highlights changes made to the original version. This file should be uploaded as separate file and labeled 'Revised Manuscript with Track Changes'.
- An unmarked version of your revised paper without tracked changes. This file should be uploaded as separate file and labeled 'Manuscript'.

Please note while forming your response, if your article is accepted, you may have the opportunity to make the peer review history publicly available. The record will include editor decision letters (with reviews) and your responses to reviewer comments. If eligible, we will contact you to opt in or out.

We look forward to receiving your revised manuscript.

Kind regards,

James G. Umen, Ph. D.
Academic Editor
PLOS ONE

**Journal Requirements:**

1. When submitting your revision, we need you to address these additional requirements.

Please ensure that your manuscript meets PLOS ONE's style requirements, including those for file naming. The PLOS ONE style templates can be found at
http://www.journals.plos.org/plosone/s/file?id=wjVg/PLOSOne_formatting_sample_main_body.pdf and http://www.journals.plos.org/plosone/s/file?id=ba62/PLOSOne_formatting_sample_title_authors_affiliations.pdf

2. We note that you have stated that you will provide repository information for your data at acceptance. Should your manuscript be accepted for publication, we will hold it until you provide the relevant accession numbers or DOIs necessary to access your data. If you wish to make changes to your Data Availability statement, please describe these changes in your cover letter and we will update your Data Availability statement to reflect the information you provide.

**Comments to the Author**

1. Is the manuscript technically sound, and do the data support the conclusions?

The manuscript must describe a technically sound piece of scientific research with data that supports the conclusions. Experiments must have been conducted rigorously, with appropriate controls, replication, and sample sizes. The conclusions must be drawn appropriately based on the data presented.

Reviewer #1: Yes

Reviewer #2: Yes

2. Has the statistical analysis been performed appropriately and rigorously?

Reviewer #1: Yes

Reviewer #2: Yes

3. Have the authors made all data underlying the findings in their manuscript fully available?

The [PLOS Data policy](http://www.plosone.org/static/policies.action#sharing) requires authors to make all data underlying the findings described in their manuscript fully available without restriction, with rare exception (please refer to the Data Availability Statement in the manuscript PDF file). The data should be provided as part of the manuscript or its supporting information, or deposited to a public repository. For example, in addition to summary statistics, the data points behind means, medians and variance measures should be available. If there are restrictions on publicly sharing data—e.g. participant privacy or use of data from a third party—those must be specified.

Reviewer #1: Yes

Reviewer #2: Yes

4. Is the manuscript presented in an intelligible fashion and written in standard English?

PLOS ONE does not copyedit accepted manuscripts, so the language in submitted articles must be clear, correct, and unambiguous. Any typographical or grammatical errors should be corrected at revision, so please note any specific errors here.

Reviewer #1: Yes

Reviewer #2: Yes

5. Review Comments to the Author

Please use the space provided to explain your answers to the questions above. You may also include additional comments for the author, including concerns about dual publication, research ethics, or publication ethics. (Please upload your review as an attachment if it exceeds 20,000 characters)

Reviewer #1: This manuscript describes the isolation of two volvocine green algal species from Lake Victoria, a novel species, Eudorina compacta, and the first isolation of Colemanosphaera charkowiensis in Africa. The manuscript presents novel research, which is worthy of publication, and is clearly and concisely written. The methods are rigorous and the images are excellent. I have one suggestion meant to improve the manuscript- both the abstract and introduction overemphasize that Lake Victoria is an "ancient" lake. The introduction would be more convincing if the emphasis on ancient lakes was reduced and the value of characterizing species ranges and cataloguing global/African biodiversity was emphasized more. Similarly, the previously known species range of C. charkowiensis should be discussed in the Introduction.
**Responses: In order to explain the significance of the collection site being an ancient lake, the following three references ([6-8]) suggesting the importance of ancient lakes worldwide in the biodiversity studies have been cited in the Introduction section of the revised manuscript.**

**6. Martens K. Speciation in ancient lakes. Trends Ecol Evol. 1997; 12: 177–182. doi: 10.1016/s0169-5347(97)01039-2.**

**7. Cristescu ME, Adamowicz SJ, Vaillant JJ, Haffner DG. Ancient lakes revisited: from the ecology to the genetics of speciation. Mol Ecol. 2010; 19: 4837–4851. doi: 10.1111/j.1365-294X.2010.04832.x.**

**8. Kulikovskiy MS, Lange-Bertalot H, Kuznetsova IV. Lake Baikal: hotspot of endemic diatoms II. In: Lange-Bertalot H. editor. Iconographia Diatomologica Volume: 26. Oberreifenberg: Koeltz Scientific Books; 2015. pp. 1-656.**

**Species range of *C. charkowiensis* had been discussed in the Results and Discussion section; it is not needed to discuss in the Introduction section.**

Minor comment:
Page 5, second to last line of "Molecular analyses" section: "stains" should be "strains".
**Response: Done as suggested.**

Reviewer #2: In the submitted manuscript, the authors described two colonial volvocalean green algae isolated from Lake Victoria (Tanzania). The ms is well written and all results are sound. The phylogenetic analyses are state-of-the-art. However, I have two major concerns.
1. The story is very small and is only of interests for small community. Therefore, I don't know if that should be published in a journal of wider audience such as PLOS One, but I leave this point to the editor.

**Response: PLoS ONE does not discriminate based on perceived impact or target audience size, so no need to address this comment**. 
2. Many studies about volvocalean algae have demonstrated that most genera are not monophyletic. So, the genus Eudorina is split at least into three lineages as the authors also showed. Therefore, in my view it is not possible to describe a new species of Eudorina if the status of this genus is not resolved. Even the type species of Eudorina, E. elegans, appears on different position in the phylogenetic analyses. It is clear that the new strain represents an own species, but if the authors want to describe a new species, the status of Eudorina elegans and the generic concept of Eudorina should be resolved. This can be done if the authors decide which strain of E. elegans will be designated as epitype. The epitype strain should be an isolate from Germany, near Berlin (type locality).

**Response: The main object of the present study is to delineate a new species of *Eudorina*. Although the polyphyly of the genus *Eudorina* has been a serious taxonomic problem, many further data of sequences and unobserved morphology are needed. Thus, the generic concept of the genus *Eudorina* has not been revised in this manuscript.**

6. PLOS authors have the option to publish the peer review history of their article ([what does this mean?](https://journals.plos.org/plosone/s/editorial-and-peer-review-process#loc-peer-review-history)). If published, this will include your full peer review and any attached files.


If you choose “no”, your identity will remain anonymous but your review may still be made public.


**Do you want your identity to be public for this peer review?** For information about this choice, including consent withdrawal, please see our [Privacy Policy](https://www.plos.org/privacy-policy).

Reviewer #1: No

Reviewer #2: No


[NOTE: If reviewer comments were submitted as an attachment file, they will be attached to this email and accessible via the submission site. Please log into your account, locate the manuscript record, and check for the action link "View Attachments". If this link does not appear, there are no attachment files to be viewed.]


While revising your submission, please upload your figure files to the Preflight Analysis and Conversion Engine (PACE) digital diagnostic tool, <https://pacev2.apexcovantage.com/>. PACE helps ensure that figures meet PLOS requirements. To use PACE, you must first register as a user. Registration is free. Then, login and navigate to the UPLOAD tab, where you will find detailed instructions on how to use the tool. If you encounter any issues or have any questions when using PACE, please email us at [figures@plos.org](mailto:figures@plos.org). Please note that Supporting Information files do not need this step.

*In compliance with data protection regulations, you may request that we remove your personal registration details at any time.* [*(Remove my information/details)*](https://www.editorialmanager.com/pone/login.asp?a=r)*. Please contact the publication office if you have any questions.*
